# Facilitation of Definitive Cancer Diagnosis With Quantitative Molecular Assays of *BRAF* V600E and *TERT* Promoter Variants in Patients With Thyroid Nodules

**DOI:** 10.1001/jamanetworkopen.2023.23500

**Published:** 2023-07-28

**Authors:** Guodong Fu, Ronald S. Chazen, Eric Monteiro, Allan Vescan, Jeremy L. Freeman, Ian J. Witterick, Christina MacMillan

**Affiliations:** 1 Alex and Simona Shnaider Research Laboratory in Molecular Oncology, Lunenfeld-Tanenbaum Research Institute, Mount Sinai Hospital, Sinai Health, Toronto, Ontario, Canada; 2 Department of Otolaryngology–Head & Neck Surgery, Mount Sinai Hospital, Sinai Health and University of Toronto, Toronto, Ontario, Canada; 3 Joseph and Mildred Sonshine Family Centre for Head and Neck Diseases, Mount Sinai Hospital, Sinai Health, Toronto, Ontario, Canada; 4 Department of Pathology and Laboratory Medicine, Mount Sinai Hospital, Sinai Health and University of Toronto, Toronto, Ontario, Canada

## Abstract

**Importance:**

Molecular testing of the presence of pathogenic genomic variants in a tumor without quantifying the variant allele fraction (VAF) does not differentiate the variation extent among tumors, often resulting in an inconclusive diagnosis because of interpatient variability.

**Objective:**

To examine the association between the quantification of VAFs of *BRAF* V600E and *TERT* promoter variants and a definitive cancer diagnosis of thyroid tumors.

**Design, Setting, and Participants:**

This diagnostic study analyzed a cohort of 378 surgically resected thyroid tumors with a maximum dimension of 1 cm or larger between March 15, 2016, and March 16, 2020, and a separate cohort of 217 residual thyroid fine-needle aspiration (FNA) biopsy specimens obtained from January 22, 2020, to March 2, 2021, at Mount Sinai Hospital, Toronto, Ontario, Canada. Data analysis was conducted between February 1, 2021, and February 1, 2023.

**Exposures:**

Quantitative VAF assays of *BRAF* V600E and *TERT* promoter variants (C228T and C250T) were performed by digital polymerase chain reaction molecular assays.

**Main Outcomes and Measures:**

The VAFs of *BRAF* V600E and *TERT* promoter variants were correlated with tumor histologic diagnoses and histopathologic features to delineate the association of VAF assays with tumor malignancy. The receiver operating characteristic curve analysis, sensitivity, specificity, positive predictive value, negative predictive value, and logistic regression analysis based on follow-up histopathologic types were used to determine the diagnostic utility of the quantitative molecular assays.

**Results:**

A total of 595 specimens, including 378 surgically resected thyroid tumors and 217 thyroid nodule FNA biopsy specimens, were collected from 580 patients (436 [75.2%] female with a mean [SD] age of 50 [16] years and 144 [24.8%] male with a mean [SD] age of 55 [14] years). Sensitive VAF assays of 378 thyroid tumors revealed the presence of the *BRAF* V600E variant in 162 tumors (42.9%), with 26 (16.0%) at a low VAF of 1% or less and 136 (84.0%) at a high VAF of greater than 1%, and the presence of *TERT* promoter variants in 49 tumors (13.0%), including 45 C228T variants (91.8%), 15 (33.3%) of which were quantified as having a low VAF (≤1%) and 30 (66.7%) as having a high VAF (>1%), and 4 C250T variants (8.2%) with VAFs between 40.0% and 47.0%. All tumors detected with *BRAF* V600E and/or *TERT* promoter variants, whether at low or high VAFs, received a definitive cancer diagnosis. Further analysis delineated a significant association between high VAFs of either variant individually or different VAF levels for both variants in coexistence and aggressive histopathologic features of tumors. Excluding low VAFs assisted in identifying patients at an intermediate-to-high risk of recurrence (odds ratio, 5.3; 95% CI, 1.9-14.6; *P* = .001). The VAF assays on the residual FNA biopsy specimens showed a high agreement to those on surgical tissues (κ = 0.793, *P* < .001) and stratified malignancy in 40 of 183 indeterminate FNA cases (21.9%), with a sensitivity of 93.8% (95% CI, 67.7%-99.7%), specificity of 90.0% (95% CI, 75.4%-96.7%), positive predictive value of 78.9% (95% CI, 53.9%-93.0%), and negative predictive value of 97.3% (95% CI, 84.2%-99.9%).

**Conclusions and Relevance:**

This diagnostic study suggests that sensitive quantitative VAF assays of *BRAF* V600E and *TERT* promoter variants can elucidate the interpatient variability in tumors and facilitate a definitive cancer diagnosis of thyroid nodules by differentiating the variation extent of genomic variants, even at low VAFs.

## Key Points

**Question:**

Does quantitative molecular assay of variant allele fraction (VAF) of genomic variants facilitate cancer diagnosis among patients with thyroid nodules?

**Findings:**

In this diagnostic study of 378 surgically resected thyroid tumors, sensitive VAF molecular assays were established to elucidate interpatient variability and variation extent of *BRAF* V600E and *TERT* promoter variants in thyroid tumors. All tumors harboring either of these variants, whether at low or high VAFs, were diagnosed histopathologically as malignant, and high VAFs of either variant alone or different VAF levels for both variants in coexistence were associated with aggressive histopathologic features and intermediate to high risks of recurrence.

**Meaning:**

Findings of this study suggest that sensitive VAF assays can quantitatively detect *BRAF* V600E and *TERT* promoter variants in thyroid tumors and facilitate a definitive diagnosis of cancer among thyroid nodules by elucidating interpatient variability of oncogenic gene variants in tumors.

## Teaser

This diagnostic study examines the association of variant allele fractions of *BRAF* V600E and *TERT* promoter variants with tumor malignancy and the clinical utility of variant allele fraction assays in assisting with definitive cancer diagnoses among patients with thyroid nodules.

## Introduction

Thyroid nodules are common clinical findings. Most thyroid nodules are benign, and only 7% to 15% are ultimately diagnosed as cancer^1^; however, thyroid cancer incidences have been increasing substantially worldwide, largely in papillary thyroid cancer (PTC).^2,3,4^ The ability to differentiate malignant from benign nodules at the time of fine-needle aspiration (FNA) can facilitate appropriate treatment for patients with cancer. Cancer arises from genomic alterations that can lead to dysregulation of gene expression and drive oncogenesis within and across tumor types.^5,6,7,8,9,10^ Significant advances have been made in the understanding of cancer molecular pathogenesis, including genetic and epigenetic alterations.^8,11^ Molecular assays, such as real-time polymerase chain reaction (PCR) and next-generation sequencing (NGS), have arisen to detect genomic changes in tumors as novel cancer diagnostic approaches with great potential in the preoperative stratification of malignancy or benignity in thyroid nodules.^12,13,14,15^ Most cancer gene variants in most patients occur at intermediate frequencies (2%-20%) or lower.^16^ Tumors harboring the same genomic variant at different levels may not behave the same among patients. Hence, molecular testing of the presence of pathogenic genomic variants in a tumor without quantifying the variant allele fraction (VAF), while offering a risk prediction, often results in an inconclusive diagnosis or adds limited benefits to treatment decision-making due to interpatient variability.^17,18^

*BRAF* (OMIM 164757) variation within the protein-coding region by an alteration of thymine (T) to adenine (A) at nucleotide position 1799 in exon 15 (*BRAF* T1799A) results in a valine (V) to glutamic acid (E) substitution at residue 600 of the BRAF protein (BRAF V600E), leading to constitutive activation of mitogen-activated protein kinase signaling in tumor development.^5,19^ Telomerase reverse transcriptase gene (*TERT*; OMIM 187270) variations occur at 2 hot spots within the gene regulatory region by transversions of cytosine (C) to thymine (T) at chromosome 5: 1 295 228C (C228T) and chr5:1,295,250C (C250T), corresponding to position −124 and −146 base pair in the proximal *TERT* gene promoter, upstream from the translational start site ATG. *TERT* promoter variants play oncogenic roles in human cancers, partially because the 2 recurrent variations create a consensus binding site for ETS transcription factor.^6,7,20^ Previous studies^8,21,22,23,24,25,26^ have shown clinical potential of *BRAF* V600E and *TERT* promoter variants in serving as molecular markers, alone or in panels, for thyroid cancer risk prediction. However, the association of interpatient variability of these genetic variants with tumor histopathology has not been well established in both research and clinic settings.^27,28,29,30,31,32^ In addition, lower variant events are likely missing without highly sensitive detection methods. In this study, we examined the association of VAFs of *BRAF* V600E and *TERT* promoter variants with tumor malignancy and assessed the clinical utility of VAF assays in assisting with definitive cancer diagnoses among patients with thyroid nodules.

## Methods

This diagnostic study was conducted in accordance with the Standards for Reporting of Diagnostic Accuracy (STARD) reporting guideline under review and approval by the Sinai Health Research Ethics Board. All participants provided written informed consent, and all patient data were deidentified.

### Patients and Specimens

Patients (n = 580) were prospectively recruited and enrolled in this study at Mount Sinai Hospital, Sinai Health, a University of Toronto–affiliated hospital and a prime referral center in Toronto, Ontario, Canada. A total of 414 fresh tissue specimens were consecutively collected from surgically resected thyroid tumors and snap-frozen at −80 °C between March 15, 2016, and March 16, 2020 (eFigure in Supplement 1). Among them, 378 tumors with a maximum dimension of 1 cm or larger were included in the study, whereas tumors smaller than 1 cm were excluded because they were identified as microcarcinomas. A separate cohort of thyroid FNA biopsy specimens was also consecutively obtained for a validation test. After routine cytologic diagnosis according to the Bethesda System for Reporting Thyroid Cytopathology^33^ and subsequent exclusion of benign cytologic results, 217 residual FNA materials were collected from 202 patients and stored at −20 °C, as previously described,^34^ at Mount Sinai Hospital from January 22, 2020, to March 2, 2021. Benign cytology (Bethesda category II) is a reassuring diagnosis because it can identify most nodules as benign. As a result, it is typically considered a less pressing concern in terms of immediate management. On the other hand, the atypical indeterminate FNA (Bethesda category I, III-V) is a significant focus in clinical care. This category often necessitates further testing, such as molecular assays, to determine whether the nodule requires diagnostic surgery or can be managed via active surveillance.^13,14,33^ Hence, the 217 FNA specimens collected comprised mostly indeterminate (Bethesda category I, III-V) and some malignant FNA biopsy specimens (Bethesda category VI) but no benign cytology specimens. The final histopathologic diagnoses of resected tumors were made in accordance with the protocols of the World Health Organization^35^ and the College of American Pathologists.^36^ Patients with cancer were further classified as having low, intermediate, or high risk of recurrence based on 2015 American Thyroid Association Management Guidelines for Adult Patients With Thyroid Nodules and Differentiated Thyroid Cancer.^1^ The study was performed double-blinded; neither the personnel involved in molecular analysis nor the pathologists were aware of the histopathologic characteristics associated with samples and the molecular analysis results, respectively. Information on participants’ race and ethnicity was not collected because such data were outside the scope of the study.

### Quantitative Molecular Assays for *BRAF* V600E and *TERT* Promoter Variants by Digital PCR

Quantitative molecular assay for VAF of the *BRAF* V600E variant was performed by locked nucleic acid (LNA) probe–based droplet digital PCR (dPCR) according to the recently established procedure.^34^ With the same strategy, sensitive molecular assays were developed to detect and quantify VAFs of 2 *TERT* promoter variations (C228T and C250T) using LNA probe–based dPCR with a mean (SD) limit of detection at 0.03 (0.01) copies/μL via a single test. The procedures of quantitative molecular assays for both variants and hematoxylin and eosin staining are described in the eMethods in Supplement 1.

### Statistical Analysis

Data analysis was conducted between February 1, 2021, and February 1, 2023. Diagnoses of benign nodules and noninvasive follicular thyroid neoplasms with papillary-like nuclear features (NIFTPs), which represent a tumor type having a low risk of malignant behavior,^37,38,39^ were set as the reference standard to distinguish from cancers. The diagnostic utility of the quantitative assays of VAFs of *BRAF* and *TERT* variants was assessed by the area under the curve (AUC) of the receiver operating characteristic curve at an appropriate cutoff between malignant and benign nodules. Sensitivity, specificity, positive predictive value (PPV), and negative predictive value (NPV) were calculated with 95% Wilson CIs. Associations of VAFs of *BRAF* and *TERT* variants with tumor characteristics were compared using 2-sided Pearson χ^2^ or Fisher exact tests for categorical variables and one-way analysis of variance tests for parametric continuous measures. Logistic regression analysis was performed to assess the diagnostic value of *BRAF* and *TERT* variant assays in identifying patients at an intermediate-to-high risk of recurrence. The odds ratios (ORs) were reported along with their 95% CIs. Analyses were conducted using SPSS, version 22.0 (IBM Inc), and statistical significance was based on 2-sided *P* < .05.

## Results

### Baseline Characteristics of Thyroid Nodules

A total of 595 specimens, including 378 surgically resected thyroid tumors and 217 thyroid nodule FNA biopsy specimens, were collected from 580 patients (436 [75.2%] female with a mean [SD] age of 50 [16] years and 144 (24.8%) male with a mean [SD] age of 55 [14] years). The cohort of 378 thyroid tumors included diagnoses of 58 benign tumors (15.3%), 7 NIFTP (1.9%), and 313 malignant tumors (82.8%) (Table 1; eTable 1 in Supplement 1). Among malignant tumors, 298 were PTCs, including 188 classic (63.1%), 65 follicular (21.8%), and 45 tall-cell, hobnail, and/or columnar cell variants (15.1%). The separate cohort of 217 FNA biopsy specimens comprised 34 malignant FNA specimens (15.7%) and 183 indeterminate FNA specimens (84.3%) that contained 83 cases of nondiagnostic or unsatisfactory (45.4%), 83 cases of atypia of undetermined significance or follicular lesion of undetermined significance (45.5%), and 17 cases suspicious for malignancy (9.3%) (eTable 2 in Supplement 1).

**Table 1.** Association of VAFs of *BRAF* V600E and *TERT* Promoter Variants With Histopathologic Features of Thyroid Tumors^a^

Characteristic	Patients, No. (%)	VAF of *BRAF* V600E variant				VAF of *TERT* variants (C228T and C250T)				
		VAF = 0	0.02 ≤ VAF ≤ 1	VAF > 1	*P* value	VAF = 0	0.06 ≤ VAF ≤ 1	VAF > 1	*P* value
Total No. (%)	378 (100)	216 (57.1)	26 (6.9)	136 (36.0)		329 (87.0)	15 (4.0)	34 (9.0)	
Sex									
Female	281 (74.3)	166 (59.1)	18 (6.4)	97 (34.5)	.38	247 (87.9)	11 (3.9)	23 (8.2)	.61
Male	97 (25.7)	50 (51.5)	8 (8.2)	39 (40.2)		82 (84.5)	4 (4.1)	11 (11.3)	
Age at diagnosis, mean (SD), y	49.4 (15.0)	49.6 (15.5)	45.2 (14.6)	49.7 (14.3)	.35	49.0 (14.6)	42.3 (13.2)	56.0 (17.7)	.006
<55	239 (63.2)	128 (53.6)	19 (7.9)	92 (38.5)	.17	211 (88.3)	13 (5.4)	15 (6.3)	.01
≥55	139 (36.8)	88 (63.3)	7 (5.0)	44 (31.7)		118 (84.9)	2 (1.4)	19 (13.7)	
Thyroidectomy									
Partial	135 (35.7)	84 (62.2)	13 (9.6)	38 (28.1)	.03	122 (90.4)	7 (5.2)	6 (4.4)	.05
Total	243 (64.3)	132 (54.3)	13 (5.3)	98 (40.3)		207 (85.2)	8 (3.3)	28 (11.5)	
Tumor size, cm^b^									
Mean (SD)	3.0 (2.0)	3.4 (2.1)	3.1 (2.7)	2.3 (1.3)	<.001	2.9 ( 1.9)	2.6 (1.3)	4.0 (2.5)	.007
1-2	154 (41.7)	66 (42.9)	12 (7.8)	76 (49.4)	<.001	138 (89.6)	7 (4.5)	9 (5.8)	.15
2-4	135 (36.6)	79 (58.5)	7 (5.2)	49 (36.3)		118 (87.4)	5 (3.7)	12 (8.9)	
>4	80 (21.7)	62 (77.5)	7 (8.8)	11 (13.8)		64 (80.0)	3 (3.8)	13 (16.3)	
Histologic type									
Benign	58 (15.3)	58 (100)	0	0	<.001	58 (100)	0	0	.01
NIFTP	7 (1.9)	7 (100)	0	0		7 (100)	0	0	
PTC	298 (78.8)	140 (47.0)	23 (7.7)	135 (45.3)		253 (84.9)	15 (5.0)	30 (10.1)	
FTC	10 (2.6)	9 (90.0)	0	1 (10.0)		7 (70.0)	0	3 (30.0)	
ATC	2 (0.5)	1 (50.0)	1 (50.0)	0		1 (50.0)	0	1 (50.0)	
MTC	3 (0.8)	1 (33.3)	2 (66.7)	0		3 (100)	0	0	
PTC variant^c^									
Classic	188 (63.1)	87 (46.3)	9 (4.8)	92 (48.9)	<.001	165 (87.8)	9 (4.8)	14 (7.4)	<.001
Follicular	65 (21.8)	50 (76.9)	10 (15.4)	5 (7.7)		61 (93.8)	2 (3.1)	2 (3.1)	
Tall-cell, hobnail, or columnar cell^d^	45 (15.1)	3 (6.7)	4 (8.9)	38 (84.4)		27 (60.0)	4 (8.9)	14 (31.1)	
Angioinvasion^e^									
Not identified	219 (82.6)	104 (47.5)	16 (7.3)	99 (45.2)	.28	189 (86.3)	10 (4.6)	20 (9.1)	.02
Present	46 (17.4)	27 (58.7)	4 (8.7)	15 (32.6)		34 (73.9)	1 (2.2)	11 (23.9)	
Lymphatic invasion^e^									
Not identified	167 (63.0)	101 (60.5)	14 (8.4)	52 (31.1)	<.001	142 (85.0)	6 (3.6)	19 (11.4)	.76
Present	98 (37.0)	30 (30.6)	6 (6.1)	62 (63.3)		81 (82.7)	5 (5.1)	12 (12.2)	
Perineural invasion^e^									
Not identified	283 (91.3)	149 (52.7)	24 (8.5)	110 (38.9)	<.001	249 (88.0)	14 (4.9)	20 (7.1)	<.001
Present	27 (8.7)	2 (7.4)	1 (3.7)	24 (88.9)		13 (48.1)	1 (3.7)	13 (48.1)	
Lymph node metastasis^e^									
Not identified	211 (67.4)	122 (57.8)	21 (10.0)	68 (32.2)	<.001	182 (86.3)	10 (4.7)	19 (9.0)	.33
Present	102 (32.6)	29 (28.4)	5 (4.9)	68 (66.7)		82 (80.4)	5 (4.9)	15 (14.7)	
Extrathyroidal extension^e^									
Not identified	277 (88.5)	142 (51.3)	25 (9.0)	110 (39.7)	.001	239 (86.3)	14 (5.1)	24 (8.7)	.006
Present	36 (11.5)	9 (25.0)	1 (2.8)	26 (72.2)		25 (69.4)	1 (2.8)	10 (27.8)	
AJCC stage									
Not identified	65 (17.2)	65 (100)	0	0	<.001	65 (100)	0	0	<.001
I	276 (73.0)	140 (50.7)	23 (8.3)	113 (40.9)		243 (88.0)	13 (4.7)	20 (7.2)	
II	31 (8.2)	10 (32.3)	2 (6.5)	19 (61.3)		19 (61.3)	2 (6.5)	10 (32.3)	
III	6 (1.6)	1 (16.7)	1 (16.7)	4 (66.7)		2 (33.3)	0	4 (66.7)	

Abbreviations: AJCC, American Joint Committee on Cancer; ATC, anaplastic thyroid carcinoma; FTC, follicular thyroid carcinoma; MTC, medullary thyroid carcinoma; NIFTP, noninvasive follicular thyroid neoplasm with papillary-like nuclear features; PTC, papillary thyroid carcinoma; VAF, variant allele fraction.

^a^ Data are presented as number (percentage) of cases unless otherwise indicated.

^b^ Analyses of tumor size with 369 tumor cases because of incomplete information for the rest of the cases.

^c^ Analyses of PTC variants with 298 PTC tumors.

^d^ Included 32 tall-cell, 9 hobnail, and 4 columnar cell variants.

^e^ Analyses of 265 malignant tumors for angioinvasion and lymphatic invasion, 310 for perineural invasion, and 313 for lymph node metastasis and extrathyroidal extension.

### Performance of Quantitative VAF Assays of *BRAF* V600E and *TERT* Promoter Variants in Thyroid Nodules

**Figure 1.**
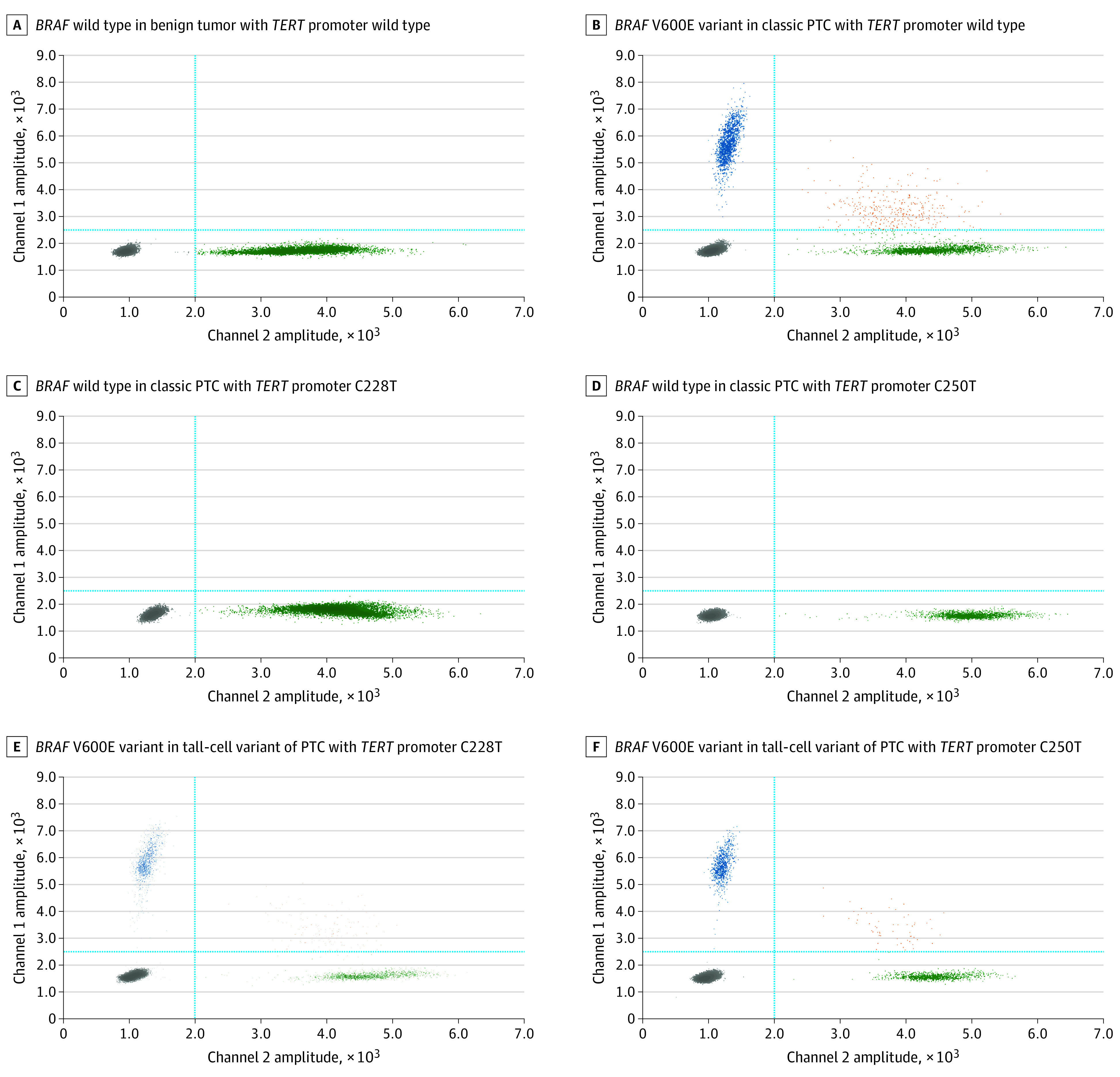
Quantitative Molecular Assay for Variant Allele Fraction (VAF) of *BRAF* V600E in Thyroid Tumors With Various Diagnoses Two-dimensional (2D) fluorescence amplitude plots and fractional abundance profiles by digital polymerase chain reaction (dPCR) assay of the *BRAF* V600E variant in thyroid tumors were presented. A, *BRAF* wild type in benign tumor with *TERT* promoter wild type; B, *BRAF* V600E variant (VAF = 47.8%) in classic papillary thyroid carcinoma (PTC) with *TERT* promoter wild type; C, *BRAF* wild type in classic PTC with *TERT* promoter C228T; D, *BRAF* wild type in classic PTC with *TERT* promoter C250T; E, *BRAF* V600E variant (VAF = 45.1%) in tall-cell variant of PTC with *TERT* promoter C228T; and F, *BRAF* V600E variant (VAF = 46.5%) in tall-cell variant of PTC with *TERT* promoter C250T. The 2D plot in each panel was from a dPCR assay of *BRAF* V600E in different specimens. The y-axis represents the variant signals via 6-fluorescein amidite probe, whereas the x-axis represents the wild-type signals via hexachloro-fluorescein probe. A cross-threshold (blue lines) was established at 2500 of channel 1 and 2000 of channel 2 to classify positive and negative droplets. Positive droplets in channel 1 (blue dots) represent the *BRAF* V600E variant, positive droplets in channel 2 (green dots) represent the *BRAF* wild type, and positive droplets in both channel 1 and channel 2 (orange dots) represent the concurrent presence of *BRAF* V600E variant and wild type, whereas double-negative droplets are presented in gray (gray dots).

**Figure 2.**
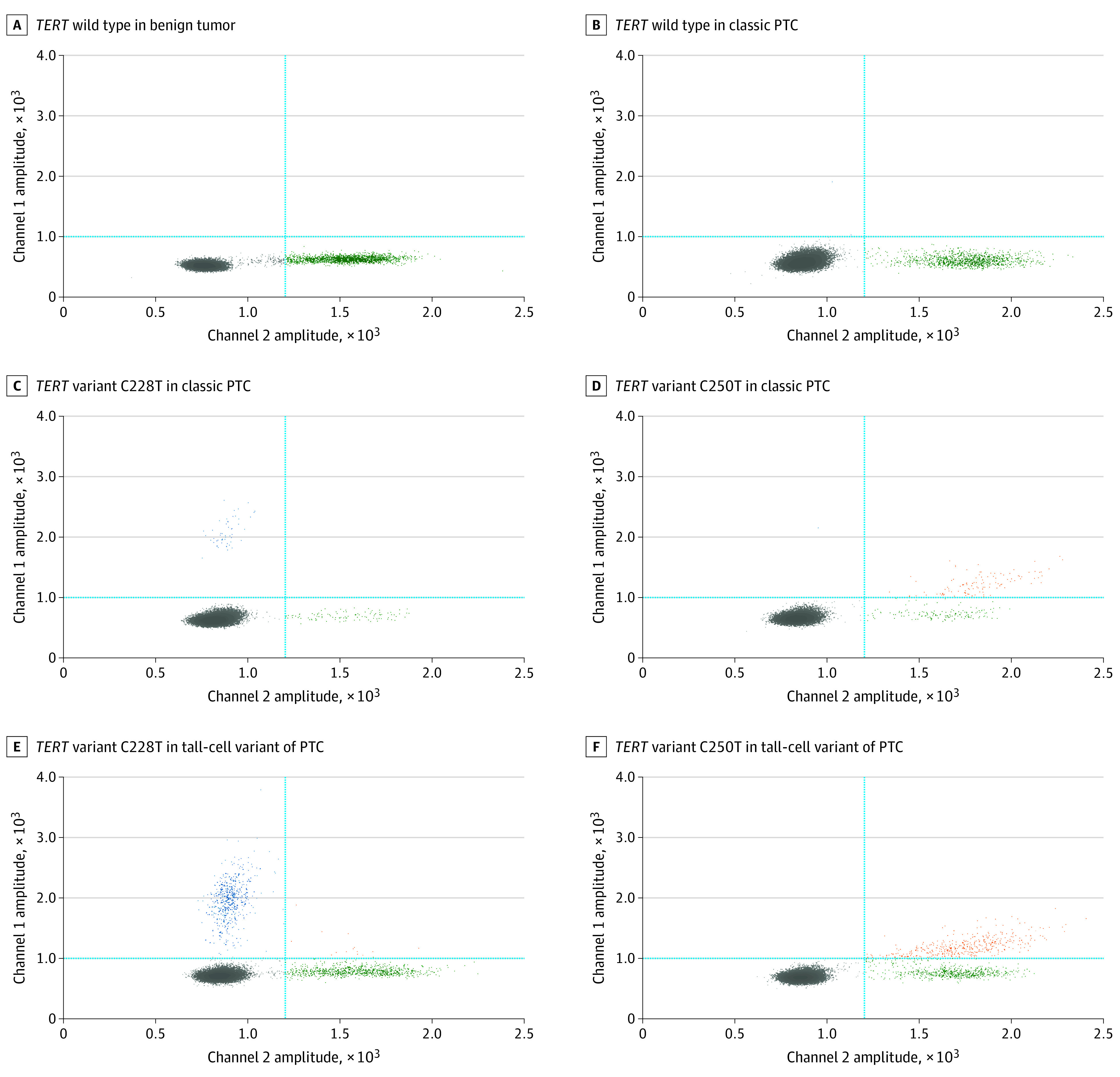
Quantitative Molecular Assay for Variant Allele Fraction (VAF) of *TERT* Promoter Variants (C228T and C250T) in Thyroid Tumors With Various Diagnoses Two-dimensional (2D) fluorescence amplitude plots and fractional abundance profiles by digital PCR (dPCR) assays of *TERT* promoter variants (C228T and C250T) in thyroid tumors were presented. A, *TERT* wild type in benign tumor; B, *TERT* wild type in classic papillary thyroid carcinoma (PTC); C, *TERT* variant C228T (VAF = 54.7%) in classic PTC; D, *TERT* variant C250T (VAF = 40.0%) in classic PTC; E, *TERT* variant C228T (VAF = 47.2%) in tall-cell variant of PTC; and F, *TERT* variant C250T (VAF = 43.0%) in tall-cell variant of PTC. The 2D plot in each panel was from dPCR assay of *TERT* promoter variants in the same sample used for the dPCR assay of *BRAF* V600E shown in the corresponding panel in Figure 1. The y-axis represents the variant signals via 6-fluorescein amidite probe, whereas the x-axis represents the wild-type signals via hexachloro-fluorescein probe. A cross-threshold (blue lines) was established at 1200 of channel 1 and 1000 of channel 2 to classify positive and negative droplets. Positive droplets in channel 1 (blue dots) represent *TERT* variant C228T, positive droplets in channel 2 (green dots) represent the wild-type *TERT*, and positive droplets in both channel 1 and channel 2 (orange dots) represent the concurrent presence of the *TERT* variant C228T and wild-type *TERT*, whereas double-negative droplets are presented in gray (gray dots). In particular, the concurrent detection of positive droplets in both channel 1 and channel 2 (orange dots) and positive droplets in channel 2 (green dots) but no detection of positive droplets in channel 1 (blue dots) represent the presence of *TERT* variant C250T.

**Figure 3.**
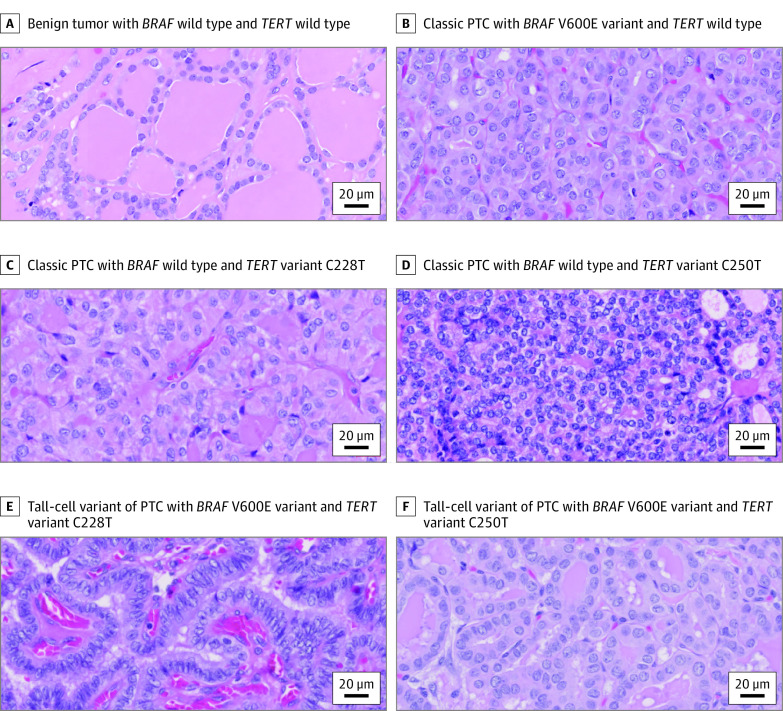
Thyroid Tumors With Various Diagnoses in Existence of Differential Expression of *BRAF* V600E and/or *TERT* Promoter Variants (C228T and C250T) Hematoxylin and eosin stain of thyroid tumors with various diagnoses was presented with the presence of *BRAF* V600E and *TERT* promoter variants that are shown in the corresponding panels of Figures 1 and 2. A, Benign tumor with *BRAF* wild type and *TERT* wild type; B, classic papillary thyroid carcinoma (PTC) with *BRAF* V600E variant (variant allele fraction [VAF] = 47.8%) and *TERT* wild type; C, classic PTC with *BRAF* wild type and *TERT* variant C228T (VAF = 54.7%); D, classic PTC with *BRAF* wild type and *TERT* variant C250T (VAF = 40.0%); E, tall-cell variant of PTC with *BRAF* V600E variant (VAF = 45.1%) and *TERT* variant C228T (VAF = 47.2%); and F, tall-cell variant of PTC with *BRAF* V600E variant (VAF = 46.5%) and *TERT* variant C250T (VAF = 43.0%). Scale = 20 μm.

Among 378 thyroid tumors, dPCR assays identified the presence of the *BRAF* V600E variant in 162 tumors (42.9%), with VAFs ranging from 0.03% to 48.6%, and *TERT* promoter variants (C228T and C250T) in 49 tumors (13.0%), with VAFs ranging from 0.13% to 54.7%, including 40 tumors (10.6%) with coexisting *BRAF* and *TERT* variants (Table 1; eTable 1 in Supplement 1). All tumors harboring *BRAF* V600E and *TERT* promoter variants at different VAF levels alone or in coexistence received a histopathologic diagnosis of malignant (Figure 1, Figure 2, and Figure 3). Neither *BRAF* V600E nor *TERT* promoter variants were detected among nodules found to be benign or NIFTP. A VAF of 1% was used to classify variants as low or high because variants with a VAF of 1% or less were hardly identified by Sanger sequencing or immunohistochemistry staining.^34^

Of the 162 *BRAF* V600E–positive tumors, 26 (16.0%) exhibited a low VAF (≤1.0%) and 136 (84.0%) exhibited a high VAF (>1.0%). The *BRAF* V600E variant was identified in 158 of 298 PTC tumors (53.0%), including 101 of 188 classic (53.7%), 15 of 65 follicular (23.1%), 32 of 32 tall-cell (100%), 7 of 9 hobnail feature (77.8%), and 3 of 4 columnar cell (75.0%) variants. Among the 49 tumors positive for *TERT* promoter variants, 15 (30.6%) were quantified at a low VAF (≤1.0%), whereas 34 (69.4%) were quantified at a high VAF (>1.0%). A total of 45 C228T variants (91.8%) and 4 C250T variants (8.2%) were detected, and both types were mutually and exclusively expressed in tumors. Of the 45 *TERT* C228T variants, 15 (33.3%) exhibited a low VAF (≤1.0%), whereas 30 (66.7%) exhibited a high VAF (>1.0%). Specifically, *TERT* C228T variation was revealed in 1 of 10 follicular thyroid carcinomas (FTCs) (10.0%) and 43 of 298 PTC tumors (14.4%), including 23 of 188 classic (12.2%), 4 of 65 follicular (6.2%), 12 of 32 tall-cell (37.5%), 2 of 9 hobnail (22.2%), and 2 of 4 columnar cell (50.0%) variants. In contrast, the 4 *TERT* C250T variants at VAFs between 40.0% and 47.0% were present in 2 of 32 PTCs with tall-cell features (6.3%) and 2 of 10 FTCs (20.0%), one reported as oncocytic variant (now updated as Hurthle cell carcinoma) and another with small foci of poorly differentiated areas, including some necrosis. Excluding the low-VAF events, the prevalence of high-VAF variants in 298 cases of PTC was 45.3% for *BRAF *V600E, 10.1% for *TERT *promoter variants, and 7.0% for the coexistence of both variants. The *BRAF* V600E variant assay showed a 100% (95% CI, 93.0%-100%) specificity and 51.8% (95% CI, 46.1%-57.4%) sensitivity at a cutoff VAF of 0.02% under the AUC of 0.76 (95% CI, 0.71-0.81) (*P* < .001) in identifying malignant from benign tumors via receiver operating characteristic curve analysis, and the *TERT* promoter variant assay showed a 100% (95% CI, 93.0%-100%) specificity and 15.7% (95% CI, 11.9%-20.2%) sensitivity at a cutoff VAF of 0.06% under the AUC of 0.58 (95% CI, 0.51-0.65) (*P* = .047). The analysis further demonstrated a 30.1% (95% CI, 24.2%-36.8%) NPV and 100% (95% CI, 97.1%-100%) PPV for the *BRAF* V600E assay and a 19.8% (95% CI, 15.7%-24.6%) NPV and 100% (90.9%-100%) PPV for the *TERT* promoter variant assay. Both assays in combination slightly improved sensitivity to 54.6% (95% CI, 48.9%-60.2%) and NPV to 31.4% (95% CI, 25.2%-38.3%) in detection of malignant tumors (eTable 3 in Supplement 1).

### Association of Quantification of VAFs of *BRAF* V600E and *TERT* Promoter Variants With Aggressive Malignancy in Thyroid Tumors and Risk of Recurrence Among Patients

All 171 tumors detected with *BRAF* V600E and/or *TERT* promoter variants were exclusively diagnosed as malignant, predominantly PTCs (Fisher exact test statistic, 95.81; *P* < .001) (Figures 1-3; eTable 1 in Supplement 1). Among them, 122 harbored *BRAF* V600E alone with diagnoses of 120 PTCs (98.4%) and 2 medullary thyroid carcinomas (1.6%), 9 harbored *TERT* variants alone with diagnoses of 7 PTCs (77.8%) and 2 FTCs (22.2%), and 40 harbored both variants with diagnoses of 38 PTCs (95.0%), 1 FTC (2.5%), and 1 anaplastic thyroid carcinoma (2.5%). Of the 3 medullary thyroid carcinomas, 2 were identified with the presence of *BRAF* V600E alone at a low VAF (<1%) and none identified with *TERT* variants. For the 2 anaplastic thyroid carcinomas, one was detected with the coexistence of *BRAF* V600E and *TERT* variants and the other was negative for both. By univariate regression analysis, both *BRAF* V600E and *TERT* promoter variants with a higher VAF (>1%) were significantly associated with tumors undergoing total thyroidectomy (Fisher exact test estimates, 6.88 [*P* = .03] for *BRAF* and 6.13 [*P* = .05] for *TERT* variants), with the presence of extrathyroidal extension (Fisher exact test estimates, 12.99 [*P* = .001] for *BRAF* and 9.69 [*P* = .006] for *TERT* variants), with perineural invasion (Fisher exact test estimates, 26.07 [*P* < .001] for *BRAF* and 28.86 [*P* < .001] for *TERT* variants), or at American Joint Committee on Cancer/TNM stage II to III (Fisher exact test estimates, 83.26 [*P* < .001] for *BRAF* and 41.77 [*P* < .001] for *TERT* variants). The *BRAF* V600E variant at a high VAF was significantly associated with small tumors (χ^2^ = 30.72; *P* < .001), lymphatic invasion (χ^2^ = 26.38; *P* < .001), or lymph node metastases (χ^2^ = 33.19; *P* < .001), whereas *TERT* variants at a high VAF were significantly associated with patients older than 55 years (χ^2^ = 9.00; *P* = .01) or larger tumors (*F*_2_ between groups and *F*_366_ within groups = 5.04; *P* = .007) or with angioinvasion (Fisher exact test estimate, 7.15; *P* = .02). The coexistence of *BRAF* V600E and *TERT* promoter variants at different VAF levels was highly associated with PTC tumors with aggressive histopathologic features (Fisher exact test estimate, 68.78; *P* < .001), such as lymphatic invasion (Fisher exact test estimate, 24.28; *P* < .001), perineural invasion (Fisher exact test estimate, 35.87; *P* < .001), lymph node metastases (Fisher exact test estimate, 24.30; *P* < .001), extrathyroidal extension (Fisher exact test estimate, 13.01; *P* = .003), and advanced American Joint Committee on Cancer/TNM stages (Fisher exact test estimate, 104.89; *P* < .001) (Table 1; eTable 1 in Supplement 1). In multivariate logistic regression analysis, *BRAF* and *TERT* variant molecular assays in combination showed a significant additive diagnostic value in identifying patients at an intermediate-to-high risk of recurrence (OR, 5.3; 95% CI, 1.9-14.6; *P* = .001) compared with *BRAF* V600E (OR, 3.6; 95% CI, 1.5-8.6; *P* = .004) or *TERT* variant assay alone (OR, 2.9; 95% CI, 0.6-12.4; *P* = .16) (eTable 4 in Supplement 1).

### VAF Assays of *BRAF* V600E and *TERT* Promoter Variants in Thyroid FNA Biopsy Specimens

The VAF assays identified *BRAF* V600E or *TERT* promoter variants in 40 of 183 indeterminate FNA specimens (21.9%) and in 15 of 34 malignant FNA specimens (44.1%), among which 24 indeterminate FNA specimens (13.1%) and 13 malignant FNA specimens (38.2%) showed high VAFs or coexistence of 2 variants (eTable 2 in Supplement 1). During a median (IQR) follow-up of 114.0 (56.0-250.8) days, 66 patients underwent resection, including 44 with indeterminate FNA specimens and 22 with malignant FNA specimens (Table 2). Histopathologic follow-up of surgically resected thyroid tumors confirmed that the malignancy in thyroid nodules increased the probability of high-risk histopathologic features when their prior residual FNA specimens had a high VAF of either *BRAF* V600E or *TERT* promoter variants or coexistence of both variants at any VAF (Table 2). Compared with the variant status of the matched surgical specimens, the VAF assays on residual FNA specimens showed a high agreement to those on surgical tissues (κ = 0.793; *P* < .001), with a sensitivity of 93.8% (95% CI, 67.7%-99.7%), specificity of 90.0% (95% CI, 75.4%-96.7%), PPV of 78.9% (95% CI, 53.9%-93.0%), and NPV of 97.3% (95% CI, 84.2%-99.9%).

**Table 2.** Histopathologic Follow-Up of VAF Assays on the Residual FNA Biopsy Specimens From Thyroid Nodules^a^

Characteristic	Patients, No. (%)	*BRAF* V600E variant VAF				*TERT* promoter variant VAF				VAF of *BRAF* and *TERT* variants^a^			
		Negative	Low	High	*P* value^b^	Negative	Low	High	*P* value^b^	Negative	Low	High or coexistence	*P* value^b^
Total No. (%)	66 (100)	48 (72.7)	7 (10.6)	11 (16.7)		60 (90.9)	2 (3.0)	4 (6.1)		46 (69.7)	7 (10.6)	13 (19.7)	
FNA cytologic diagnosis^c^													
ND	12 (18.2)	12 (25.0)	0	0	.005	12 (20.0)	0	0	0.73	12 (26.1)	0	0	.004
AUS or FLUS	20 (30.3)	17 (35.4)	2 (28.6)	1 (9.1)		18 (30.0)	1 (50.0)	1 (25.0)		16 (34.8)	2 (28.6)	2 (15.4)	
SFM	12 (18.2)	8 (16.7)	3 (42.9)	1 (9.1)		12 (20.0)	0	0		8 (17.4)	3 (42.9)	1 (7.7)	
Malignant	22 (33.3)	11 (22.9)	2 (28.6)	9 (81.8)		18 (30.0)	1 (50.0)	1 (75.0)		10 (21.7)	2 (28.6)	10 (76.9)	
Histopathologic finding^d^													
Benign	11 (16.7)	11 (22.9)	0	0	.08	11 (18.3)	0	0	>.99	11 (23.9)	0	0	.07
Malignant	55 (83.3)	37 (77.1)	7 (100)	11 (100)		49 (81.7)	2 (100)	4 (100)		35 (76.1)	7 (100)	13 (100)	
Invasion^e^													
Not identified	31 (62.0)	24 (70.6)	5 (71.4)	2 (22.2)	.03	31 (68.9)	0	0	.005	24 (72.7)	5 (71.4)	2 (20.0)	.01
Present	19 (38.0)	10 (29.4)	2 (28.6)	7 (77.8)		14 (31.1)	2 (100)	3 (100)		9 (27.3)	2 (28.6)	8 (80.0)	

Abbreviations: AUS, atypia of undetermined significance; FLUS, follicular lesion of undetermined significance; FNA, fine-needle aspiration; ND, nondiagnostic or unsatisfactory; SFM, suspicious for malignancy; VAF, variant allele fraction.

^a^ Negative VAF indicates 0%, low VAF indicates 0.02% to 1%, and high VAF indicates greater than 1% for the *BRAF* variant. Negative VAF indicates 0%, low VAF indicates 0.06% to 1%, and high VAF indicates greater than 1% for the *TERT* promoter variants. High VAF or coexistence of 2 variants includes both cases with a VAF greater than 1% on VAF assays of *BRAF* or *TERT* variants and cases with coexistence of 2 variants.

^b^ Fisher exact test (2-sided) for categorical variables.

^c^ Analyses were based on 66 residual FNA biopsy specimens from subsequent surgery.

^d^ Histopathologic analysis was based on surgical tumors from 66 patients.

^e^ Invasion analyses, including angioinvasion, lymphatic invasion, or infiltrative invasion, were based on 50 cases of malignant tumors with exclusion of 5 cases of microcarcinormas.

## Discussion

This diagnostic study was conducted to elucidate the clinical utility of VAF assays of *BRAF* V600E and *TERT* promoter variants in achieving a definitive diagnosis of malignancy among patients with thyroid nodules greater than 1 cm. Sensitive and specific VAF assays using LNA probe–based dPCR were first developed to quantify and discriminate between 2 *TERT* promoter variants, C228T and C250T, in a single test. The study demonstrated that thyroid nodules presenting *BRAF* V600E at a VAF of 0.03% or higher and/or *TERT* promoter variants at a VAF of 0.13% or higher could receive a definitive cancer diagnosis. Further analysis delineated a significant association of high VAFs alone or different VAF levels of both variants in coexistence with aggressive histopathologic features in tumors and an intermediate-to-high risk of recurrence in patients. In addition, VAF assays of residual FNA biopsy specimens stratified malignant tumors, particularly those with unfavorable histopathologic features, among thyroid nodules with indeterminate FNA specimens.

The detection of oncogene variants at low copies or structural complexity remains a challenge even by the power-read DNA sequencing or the high-intensity NGS.^40^ The target region of *TERT* promoter variants contains up to 80% of nucleotide guanine and cytosine content, which results in a biological or technical hindrance for the detection of these variants,^23,41,42,43^ although the leading-edge LNA probe–based dPCR approach enables a highly sensitive and accurate detection and absolute quantification of variant DNA copies.^34,44^ In this study, dPCR assays were established for detecting the 2 *TERT* promoter variations (C228T and C250T) at a mean (SD) limit of detection of 0.03 (0.01) copies/μL in a single test, greatly improving the detection efficiency and sensitivity. The 2 *TERT* variants identified by dPCR assays were mutually and exclusively expressed in tumors, with a dominant expression of C228T (91.8%), consistent with others’ findings.^21^

The current study provided a comprehensive analysis of the association of VAFs of *BRAF* V600E and *TERT* promoter variants with a definitive diagnosis of tumor malignancy and histopathologic features at presentation. The VAF level of a gene deleterious variant within a tumor refers to the fraction of cells having the variant of interest and reflects the extent of pathogenesis of the tumor. It may further inform a definitive cancer diagnosis and an appropriate treatment once its association with clinicopathologic features has been established. In the current study, differential VAF profiles of the 2 variants were observed in PTC and FTC, with *BRAF* V600E being more associated with PTC (53.0%) than FTC (10.0%), and conversely, *TERT* variants being less associated with PTC (15.1%) than FTC (30.0%). In particular, high VAFs were associated with 91.1% classic, 33.3% follicular, 87.5% tall-cell, 100% hobnail feature, and 100% columnar cell variants of PTC among *BRAF* V600E–positive tumors and 60.9% classic, 50.0% follicular, 78.6% tall-cell, 100% hobnail feature, and 50.0% columnar cell variants of PTC among *TERT* variant–positive tumors. Notably, the findings of 66.7% medullary thyroid carcinomas presenting with the *BRAF* V600E variant at a low VAF (<1%) and 50% of anaplastic thyroid carcinomas coexisting with both variants need to be confirmed in a larger cohort of these lesions. Significant interpatient variability of *BRAF* and *TERT* variants was delineated within PTCs, with VAFs quantified in a wide range of 0.03% to 48.6% for *BRAF* and 0.13% to 54.7% for *TERT*. Excluding the low-VAF events, the prevalence of high-VAF variants in 298 cases of PTC was 45.3% for *BRAF* V600E, 10.1% for *TERT* promoter variants, and 7.0% for the coexistence of both variants. These rates align with the findings in The Cancer Genome Atlas study.^8^ These results indicate that the low-VAF events were previously undetected and overlooked by NGS-based testing. It is well documented that *BRAF* V600E activates the mitogen-activated protein kinase pathway and *TERT* promoter variants generate a consensus binding site for ETS transcription factors to enhance *TERT* transcriptional activity, which may lead to increased tumor cell growth in human cancers.^5,7,21,45^ The distributions of variation frequencies in different variated genes have been reported across cancer types,^46^ but the exact clinical roles of interpatient variability of oncogenic variants and their particular low-VAF events are yet to be understood. Our data revealed for the first time, to our knowledge, the occurrence of low-VAF events in 16.0% of *BRAF* V600E variant–positive tumors and 30.6% of *TERT* promoter variant–positive tumors. These low-VAF tumors shared similarity with high-VAF tumors in histopathologic diagnosis as malignant but were distinct in their classification as low risk. This result underscores the clinical significance of detecting low-VAF events that have often been missed in prior studies. Our results demonstrated that classifying tumors based on VAF assays aids in making a definitive diagnosis of thyroid cancer and determining the extent of malignancy. Tumors harboring a high VAF of *BRAF* V600E or *TERT* promoter variants alone or different VAF levels of both variants in coexistence exhibited malignancy with aggressive histopathologic features, suggesting VAF assays of FNA biopsy specimens may facilitate a preoperative evaluation of either an aggressive or indolent malignancy of indeterminate thyroid nodules. In addition, low VAFs are linked to low-risk nodules, and excluding low-VAF variants increases the association of tumors with unfavorable histopathologic findings, assisting in avoidance of overdiagnosis and overtreatment.

### Limitations

Several limitations are of concern. This analysis of a single-center cohort showed that 82.8% of thyroidectomy specimens were malignant. Further validation in prospective studies will be required to confirm the routine clinical application of VAF assays using a larger cohort of patients, including a greater sample size of various benign tumors and nontumor conditions (such as Hashimoto thyroiditis). The VAF analysis of the 2 variants in frozen tissues, mostly based on a single tumor sample, might not reflect the full clinical or genomic scope of the respective variants when there are multifocal tumors. Despite the 100% specificity and improved sensitivity of VAF assays, this study was not intended to present an ideal test with 2 markers to identify all patients with cancer because a portion of patients with cancer carry other drivers but no *BRAF* V600E and *TERT* promoter variants. Therefore, quantitative assays of additional actionable markers need to be developed for malignancy detection among these patients.

## Conclusions

In this diagnostic study, sensitive quantitative molecular assays for VAFs of *BRAF* V600E and *TERT* promoter variants were found to elucidate interpatient variability in tumors. In addition, these assays can facilitate a definitive cancer diagnosis of thyroid nodules by differentiating the variation extent of genomic variants, even at low VAFs.

## Notes

Supplement 1. **eMethods.** Supplemental Methods **eTable 1.** Association of *BRAF* V600E and *TERT* Promoter Variants Alone or in Coexistence With Histopathologic Features of Thyroid Tumors **eTable 2.** Quantification of VAFs of *BRAF *V600E and *TERT *Promoter Variants in Indeterminate FNA Biopsies From Thyroid Nodules **eTable 3.** Performance of dPCR Assays of *BRAF *V600E Alone, *TERT *Promoter Variants Alone, or Both Variants in Combination for the Detection of Malignancy in Thyroid Tumors **eTable 4.** Association of *BRAF *V600E or *TERT *Promoter Variants at High VAFs Alone or in Coexistence With Patients at an Intermediate-to-High Risk in Recurrence **eFigure.** Participant Flow Chart

Supplement 2.
Data Sharing Statement
