# Supplementary material for: Facilitation of Definitive Cancer Diagnosis With Quantitative Molecular Assays of BRAF V600E and TERT Promoter Variants in Patients With Thyroid Nodules
Source: JAMA Netw Open. 2023 Jul 28;6(7):e2323500. doi: 10.1001/jamanetworkopen.2023.23500 (PMC10383015; doi:10.1001/jamanetworkopen.2023.23500)
Supplement: Supplement 1. — eMethods. Supplemental Methods eTable 1. Association of BRAF V600E and TERT Promoter Variants Alone or in Coexistence With Histopathologic Features of Thyroid Tumors eTable 2. Quantification of VAFs of BRAF V600E and TERT Promoter Variants in Indeterminate FNA Biopsies From Thyroid Nodules eTable 3. Performance of dPCR Assays of BRAF V600E Alone, TERT Promoter Variants Alone, or Both Variants in Combination for the Detection of Malignancy in Thyroid Tumors eTable 4. Association of BRAF V600E or TERT Promoter Variants at High VAFs Alone or in Coexistence With Patients at an Intermediate-to-High Risk in Recurrence eFigure. Participant Flow Chart [file jamanetwopen-e2323500-s001.pdf]

Supplementary Online Content

Fu G, Chazen RS, Monteiro E, et al. Facilitation of definitive cancer diagnosis with quantitative molecular assays of *BRAF* V600E and *TERT* promoter variants in patients with thyroid nodules. *JAMA Netw Open*. 2023;6(7):e2323500. doi:10.1001/jamanetworkopen.2023.23500

eMethods. Supplemental Methods
eTable 1. Association of *BRAF* V600E and *TERT* Promoter Variants Alone or in Coexistence With Histopathologic Features of Thyroid Tumors
eTable 2. Quantification of VAFs of *BRAF* V600E and *TERT* Promoter Variants in Indeterminate FNA Biopsies From Thyroid Nodules
eTable 3. Performance of dPCR Assays of *BRAF* V600E Alone, *TERT* Promoter Variants Alone, or Both Variants in Combination for the Detection of Malignancy in Thyroid Tumors
eTable 4. Association of *BRAF* V600E or *TERT* Promoter Variants at High VAFs Alone or in Coexistence With Patients at an Intermediate-to-High Risk in Recurrence
eFigure. Participant Flow Chart

This supplementary material has been provided by the authors to give readers additional information about their work.

© 2023 Fu G et al. *JAMA Network Open.*

1 eMethods

1.1 Quantitative molecular assays for *BRAF* V600E and *TERT* promoter variants (C228T and C250T)

1.1.1 Thyroid cancer cells harboring the wild-type or variants of *BRAF* and *TERT* promoters

Human follicular thyroid cancer–derived cell line FTC-133 harboring the *BRAF* wild-type and *TERT* C228T variant, and papillary thyroid cancer-derived BCPAP cell harboring *BRAF* V600E variant and *TERT* wild-type (kindly provided by Dr. Shilpa Thakur, NIH, MD, USA)[1] were used as the positive or negative controls for detection of *BRAF* and *TERT* variations. FTC-133 cells were cultured in DMEM growth medium (Invitrogen, CA, USA) supplemented with 10% fetal bovine serum (FBS) and BCPAP cells in RPMI 1640 medium (Invitrogen) with 5% FBS, containing 100 U/mL penicillin and 100 mg/mL streptomycin in both mediums in a humidified 5% CO_2 incubator. The two cell lines' origin from thyroid cancers was confirmed by cell authentication via the short tandem repeat profiling analysis by TCAG Genetic Analysis Facility (The Centre for Applied Genomics, ON, CAN).

1.1.2 Genomic DNA extraction

Genomic DNA was extracted from FTC-133 and BCPAP cells, tumor specimens and residual FNA biopsies. The FTC-133 and BCPAP cells and thyroid tissue specimens were digested with 20 µL of Proteinase K (Qiagen, Hilden, Germany) at 55 °C for 1 h with mild agitation, and followed at 90 °C for an additional 1 h. Extraction of genomic DNA was carried out using the QIAamp DNA Mini Kit (Qiagen). DNA concentration was measured using NanoDrop 1000 (Thermo Fisher Scientific, MA, USA).

1.1.3 Quantitative molecular assays of *BRAF* V600E and *TERT* promoter variants (C228T and C250T) using droplet dPCR approach

Quantitative molecular assays of *BRAF* V600E and *TERT* promoter variants were performed using droplet dPCR on the QX200 AutoDG Droplet Digital PCR System (Bio-Rad Laboratories, ON, CAN) in the Central Scientific Laboratory of Lunenfeld-Tanenbaum Research Institute, Toronto, Canada. Molecular assay of *BRAF* V600E variant was conducted to quantify copies of target variant and wild-type in a linear response manner and allow for further quantification of the VAF level with BRAFV600E98 Assay (20X) according to the recently established procedure.[2] With the same strategy, molecular assay was developed to detect and quantify VAFs of *TERT* variations (C228T and C250T) using LNA probe-based dPCR with TERT Variation Assays (20x). Briefly, droplet dPCR assays were performed in a 22-µL reaction mixture containing 11 µL dPCR Supermix for Probes (2×, no dUTP) (Bio-Rad Laboratories), 1.1 µL of TERT Variation Assay (20×), 3.5 µL of Enhanced solution, 3.0 µL of genomic DNA, and 3.4 µL of deionized water. Droplets were generated using the QX200 Droplet Generator (Bio-Rad Laboratories) and subjected to thermal cycling for amplification under conditions: 1 cycle of 95 °C for 10 min, 45 cycles of 94 °C for 30 s and 54 °C for 1 min, 1 cycle of 98 °C for 10 min with a ramp rate of 2°C/s, and hold at 4 °C. Fractional abundance was acquired by the QX200 droplet reader and calculated with QuantaSoft analysis software (Bio-Rad Laboratories). The *TERT* variant allele and wild-type allele concentration in the final PCR reaction mix, presented as copies per microliter, were calculated from the values of 6-fluorescein amidite (6-FAM)-positive droplets (variants) and hexachloro-fluorescein (HEX)-positive droplets (wild-type) by applying a fluorescence amplitude threshold. The VAF was computed as percentage of the variant positive droplets of the total positive droplets of *TERT* wild-type and variants. FTC-133 DNA containing the *TERT* variant (C228T), BACAP DNA containing the *TERT* wild-type, and blank control (H_2O) were included in each test to verify the assay condition and exclude potential contamination.

1.1.4 Validation of *TERT* promoter variants by Sanger sequencing

The results of dPCR assay of *TERT* promoter variants (C228T and C250T) was further validated by power read sequencing. Briefly, the target amplicon was amplified using the NEBNext Ultra II Q5® Master Mix (New England Biolabs, MA, USA) by a nested PCR comprised of the first PCR, with forward 5'- CTTCCCACGTGCGCAGCAGGA and reverse 5'- AGTGGATTCGCGGGCACAGA primers, and the second PCR, with forward 5'- CAGCGCTGCCTGAAACTCG and reverse 5'- ACCCGTCCTGCCCCTTCACCTT primers, at an annealing temperature of 60 ºC. After purification of PCR products using the Monarch® DNA Gel Extraction Kit (New England Biolabs), Sanger sequencing was carried out on a power read mode of Applied Biosystems 3730 XL DNA Analyzers (Eurofins Genomics, KY, USA). PCR amplicons from BCPAP and FTC-133 DNA as control were included in each sequencing to verify dPCR assay results.

© 2023 Fu G et al. *JAMA Network Open.*

1.1.5 Assessment of sensitivity, specificity, positive predictive value, negative predictive value and odds ratio

The accuracy and reproducibility of dPCR assay of *TERT* promoter variants was assessed according to the procedure described previously.[2] In this study, dPCR assays were established for detecting the two *TERT* promoter variations at a mean (SD) limit of detection 0.03 (0.01) copies/μL in a single test, greatly improving the detection efficiency and sensitivity. Logistic regression analysis was performed to assess the diagnostic value of *BRAF* and *TERT* variant assays in identifying patients at an intermediate-to-high risk of recurrence. The clinical sensitivity, specificity, and positive predictive values, negative predictive values and odds ratios with 95% CIs of *BRAF* V600E and *TERT* promoter variations for tumor malignancy were calculated using standard formulas in accordance with the Standards for Reporting of Diagnostic Accuracy (STARD) reporting guidelines.[3]

1.2 Hematoxylin and Eosin staining

Hematoxylin and Eosin (H&E) staining was performed using FFPE tissue sections (4 μm) in Mount Sinai Services (Mount Sinai Hospital, ON, CAN). Briefly, after removal of the wax on the tissue with xylene and hydration via several changes of alcohol, the sections were subjected to a nuclear stain with Harris hematoxylin followed by an eosin counterstain. After passing serials of alcohol to remove all traces of water, the slide was rinsed in several baths of xylene which clears the tissue. A glass coverslip was mounted on a thin layer of polystyrene mountant on the tissue. H&E histology slides were scanned by Microscopy Slide Scanner ZEISS Axio Scan.Z1 (Zeiss, Oberkochen, Germany) in the OPTical IMAging (OPTIMA) Facility at Lunenfeld-Tanenbaum Research Institute in Mount Sinai Hospital and all H&E images were acquired via ZEISS ZEN (blue edition).

Lead contact and method availability

Further information and request for the protocol of dPCR assays should be directed to and will be fulfilled by the Lead Contact, Guodong Fu (David.Fu@sinaihealth.ca; gdfu2002@gmail.com).

References:

1. Thakur S, Daley B, Gaskins K, et al. Metformin targets Mitochondrial Glycerophosphate Dehydrogenase (mGPDH) to control Rate of Oxidative Phosphorylation and growth of thyroid cancer in vitro and in vivo. *Clinical cancer research: an official journal of the American Association for Cancer Research.* 2018.
2. Fu G, Chazen RS, MacMillan C, Witterick IJ. Development of a Molecular Assay for Detection and Quantification of the BRAF Variation in Residual Tissue from Thyroid Nodule Fine-Needle Aspiration Biopsy Specimens. *JAMA Netw Open.* 2021:4(10):e2127243.
3. Bossuyt PM, Reitsma JB, Bruns DE, et al. STARD 2015: an updated list of essential items for reporting diagnostic accuracy studies. *BMJ.* 2015:351:h5527.

© 2023 Fu G et al. *JAMA Network Open.*

2. eTables

eTable 1. Association of *BRAF* V600E and *TERT* Promoter Variants Alone or in Coexistence With Clinicohistopathologic Features of Thyroid Tumors[a]

Characteristic	Patients No. (%)	*BRAF & TERT* VAF = 0	*BRAF* alone VAF ≥ 0.03	*TERT* alone VAF ≥ 0.13	*BRAF* VAF ≥ 0.03 *TERT* VAF ≥ 0.13	*P value*[b]
Total No. (%)	378 (100)	207 (54.8)	122 (32.3)	9 (2.4)	40 (10.6)	
Sex						
Male	97 (25.7)	50 (51.5)	32 (33.0)	0	15 (15.5)	*.10*
Female	281 (74.3)	157 (55.9)	90 (32.0)	9 (3.2)	25 (8.9)	
Age at diagnosis						
Mean(SD), y	49.4 (15.0)	49.7 (15.5)	47.8 (13.0)	47.9 (16.4)	52.6 (17.8)	*.33*
< 55	239 (63.2)	123 (51.5)	88 (36.8)	5 (2.1)	23 (9.6)	*.09*
≥ 55	139 (36.8)	84 (60.4)	34 (24.5)	4 (2.9)	17 (12.2)	
Thyroidectomy						
Partial	135 (35.7)	83 (61.5)	39 (28.9)	1 (0.7)	12 (8.9)	*.16*
Total	243 (64.3)	124 (51.0)	83 (34.2)	8 (3.3)	28 (11.5)	
Tumor size, cm[c]						
Mean(SD)	3.0 (2.0)	3.3 (2.1)	2.2 (1.2)	4.6 (1.7)	3.3 (2.4)	*<.001*
1 - 2	154 (41.7)	65 (42.2)	73 (47.4)	1 (0.6)	15 (9.7)	*<.001*
2 - 4	135 (36.6)	76 (56.3)	42 (31.1)	3 (2.2)	14 (10.4)	
> 4	80 (21.7)	57 (71.3)	7 (8.8)	5 (6.3)	11 (13.8)	
Histologic type						
Benign	58 (15.3)	58 (100)	0	0	0	*<.001*
NIFTP	7 (1.9)	7 (100)	0	0	0	
PTC	298 (78.8)	133 (44.6)	120 (40.3)	7 (2.3)	38 (12.8)	
FTC	10 (2.6)	7 (70.0)	0	2 (20.0)	1 (10.0)	
ATC	2 (0.5)	1 (50.0)	0	0	1 (50.0)	
MTC	3 (0.8)	1 (33.3)	2 (66.7)	0	0	
PTC variant[d]						
Classic	188 (63.1)	83 (44.1)	82 (43.6)	4 (2.1)	19 (10.1)	*<.001*
Follicular	65 (21.8)	48 (73.8)	13 (20.0)	2 (3.1)	2 (3.1)	
Tall cell, hobnail or columnar cell[e]	45 (15.1)	2 (4.4)	25 (55.6)	1 (2.2)	17 (37.8)	
Tumor focality[d]						
Unifocal	103 (32.9)	55 (53.4)	35 (34.0)	3 (2.9)	10 (9.7)	*.23*
Multifocal	210 (67.1)	87 (41.4)	87 (41.4)	6 (2.9)	30 (14.3)	
Angioinvasion[f]						
Not identified	219 (82.6)	100 (45.7)	89 (40.6)	4 (1.8)	26 (11.9)	*.02*
Present	46 (17.4)	23 (50.0)	11 (23.9)	4 (8.7)	8 (17.4)	
Lymphatic invasion[f]						
Not identified	167 (63.0)	93 (55.7)	49 (29.3)	8 (4.8)	17 (10.2)	*<.001*
Present	98 (37.0)	30 (30.6)	51 (52.0)	0	17 (17.3)	
Perineural invasion[f]						
Not identified	283 (91.1)	140 (49.5)	109 (38.5)	9 (3.2)	25 (8.8)	*<.001*
Present	27 (8.9)	2 (7.4)	11 (40.7)	0	14 (51.9)	
Lymph node metastases[f]						
Not identified	211 (67.4)	114 (54.0)	68 (32.2)	8 (3.8)	21 (10.0)	*<.001*
Present	102 (32.6)	28 (27.5)	52 (52.9)	1 (1.0)	19 (18.6)	
Extrathyroidal extension[f]						
Not identified	277 (88.5)	133 (48.0)	106 (38.3)	9 (3.2)	29 (10.5)	*.003*
Present	36 (11.5)	9 (25.0)	16 (44.4)	0	11 (30.6)	
AJCC stage						
Not identified	65 (100)	65 (100)	0	0	0	*<.001*
I	276 (73.0)	132 (47.8)	111 (40.2)	8 (2.9)	25 (9.1)	
II	31 (8.2)	9 (29.0)	10 (32.3)	1 (3.2)	11 (35.5)	
III	6 (1.4)	1 (16.7)	1 (16.7)	0	4 (66.7)	

Notes:

Abbreviations: AJCC, American Joint Committee on Cancer; ATC, anaplastic thyroid carcinoma; FTC, follicular thyroid carcinoma; MTC, medullary thyroid carcinoma; NIFTP, noninvasive follicular thyroid neoplasm with papillary-like nuclear features; PTC, papillary thyroid carcinoma; VAF, variant allele fraction.

[a] Data are presented as number (percentage) of cases unless otherwise indicated.

[b] Two-sided Pearson $\chi 2$ or Fisher's exact test for categorical variables and 1-way analysis of variance test for independent parametric continuous measures.

[c] Analyses of 369 cases for tumor size because of incomplete information of the missing cases.

[d] Analyses of 298 cases of PTC tumors, including classic, follicular and tall-cell, hobnail or columnar cell variants.

[e] Included 32 tall-cell, 9 hobnail, and 4 columnar cell variants.

[f] Analyses of 265 malignant tumors for angioinvasion and lymphatic invasion and 313 for tumor multifocality, extrathyroidal extension, lymph node metastasis and invasion.

© 2023 Fu G et al. *JAMA Network Open.*

eTable 2. Quantification of VAFs of *BRAF* V600E and *TERT* Promoter Variants in Indeterminate FNA Biopsy Specimens From Thyroid Nodules[a]

Characteristic	Patients	*Thyroid nodule FNA biopsy specimens*				*P* value[b]
	No. (%)	ND	AUS/FLUS	SFM	Malignancy	
Total No. (%)	217 (100)	83 (38.2)	83 (38.2)	17 (7.8)	34 (15.7)	
Sex						
Male	51 (23.5)	21 (25.3)	17 (20.5)	1 (5.9)	12 (35.3)	*.11*
Female	166 (76.5)	62 (74.7)	66 (79.5)	16 (94.1)	22 (64.7)	
Age at biopsy						
Mean (SD), y	55.5 (15.7)	58.1 (14.0)	56.1 (15.7)	50.0 (16.7)	50.4 (15.7)	*.04*
< 55 y	106 (48.8)	35 (42.2)	42 (50.6)	10 (58.8)	19 (55.9)	*.41*
≥ 55 y	111 (51.2)	48 (57.8)	41 (49.4)	7 (41.2)	15 (44.1)	
VAF of *BRAF* V600E[c]						
Negative	174 (80.2)	74 (89.2)	67 (80.7)	13 (76.5)	20 (58.8)	
Low	15 (6.9)	3 (3.6)	7 (8.4)	3 (17.6)	2 (5.9)	*.002*
High	28 (12.9)	6 (7.2)	9 (10.8)	1 (5.9)	12 (35.3)	
VAF of *TERT* promoter variants[d]						
Negative	199 (91.7)	78 (94.0)	77 (92.8)	15 (88.2)	29 (85.3)	
Low	8 (3.7)	1 (1.2)	5 (6.0)	1 (5.9)	1 (2.9)	*.10*
High	10 (4.8)	4 (4.8)	1 (1.2)	1 (5.9)	4 (11.8)	
VAF of *BRAF* and *TERT* variants[e]						
Negative	162 (74.7)	69 (83.1)	63 (75.9)	11 (64.7)	19 (55.9)	
Low	18 (8.3)	4 (4.8)	8 (9.6)	4 (23.5)	2 (5.9)	*.007*
High or coexistence	37 (17.1)	10 (12.0)	12 (14.5)	2 (11.8)	13 (38.2)	

Notes:

Abbreviations: AUS, atypia of undetermined significance; FLUS, follicular lesion of undetermined significance; FNA, fine-needle aspiration; ND, nondiagnostic or unsatisfactory; SFM, suspicious for malignancy; VAF, variant allele fraction.

[a] Data are presented as number (percentage) of cases unless otherwise indicated.

[b] Two-sided Pearson $\chi 2$ or Fisher's exact test for categorical variables and 1-way analysis of variance test for independent parametric continuous measures.

[c] Negative VAF indicates 0%, low VAF indicates 0.03% to 1%, and high VAF indicates greater than 1% for the *BRAF* V600E variant.

[d] Negative VAF indicates 0%, low VAF indicates 0.13% to 1%, and high VAF indicates greater than 1% for the *TERT* promoter variants.

[e] Negative VAF indicates 0% for both variants, and low VAF indicates 0.03% to 1% for the *BRAF* V600E variant alone or 0.13% to 1% for the *TERT* promoter variants alone. The high or coexistence includes both cases with a VAF greater than 1% for *BRAF* or *TERT* variants and cases in coexistence of two variants at low or high VAFs.

© 2023 Fu G et al. *JAMA Network Open.*

eTable 3. Performance of dPCR Assays of *BRAF* V600E Alone, *TERT* Promoter Variants Alone, or Both Variants in Combination for Detection of Malignancy in Thyroid Tumors

Characteristic	*BRAF* V600E variant	*TERT* promoter variants	*BRAF* & *TERT* variants
True positive[a]	162 (42.8)	49 (12.9)	171 (45.2)
False positive[a]	151 (39.9)	264 (69.8)	142 (37.6)
True negative[a]	65 (17.2)	65 (17.2)	65 (17.2)
False negative[a]	0	0	0
Sensitivity[b]	51.8 (46.1, 57.4)	15.7 (11.9, 20.2)	54.6 (48.9, 60.2)
Specificity[b]	100 (93.0, 100)	100 (93.0, 100)	100 (93.0, 100)
PPV[b]	100 (97.1, 100)	100 (90.9, 100)	100 (97.3, 100)
NPV[b]	30.1 (24.2, 36.8)	19.8 (15.7, 24.6)	31.4 (25.2, 38.3)

Notes:

Abbreviations: PPV, positive predictive value; NPV, negative predictive value.

[a] Data are presented as number (percentage) of cases.

[b] Data are presented as percentage (95% CI).

© 2023 Fu G et al. *JAMA Network Open.*

eTable 4. Association of *BRAF* V600E or *TERT* Promoter Variants at High VAFs Alone or in Coexistence With Patients at an Intermediate-to-high Risk in Recurrence[a]

Variable	OR	95% CI for OR		*P* value
		Lower	Upper	
Sex				
Female vs. Male	0.666	0.299	1.487	*.32*
Age (years)				
≥ 55 vs. < 55	0.774	0.371	1.617	*.50*
BRAF Variant Assay[b]				
high VAF vs. low VAF	3.608	1.511	8.615	*.004*
TERT Variant Assay[c]				
high VAF vs. low VAF	2.854	0.655	12.442	*.16*
BRAF + TERT Variant Assays[d]				
high VAF or in coexistence vs. low VAF	5.302	1.919	14.644	*.001*

Notes:

Abbreviations: OR, odds ratio; 95% CI, 95% confidence interval; VAF, variant allele fraction.

[a] Data were reported as OR along with 95% CI.

[b] Low VAF indicates 0.03% to 1%, and high VAF indicates greater than 1% for the *BRAF* assay.

[c] Low VAF indicates 0.13% to 1%, and high VAF indicates greater than 1% for the *TERT* promoter variants.

[d] Low VAF indicates 0.03% to 1% for the *BRAF* V600E variant alone or 0.13% to 1% for the *TERT* promoter variants alone. The high or coexistence includes both cases with a VAF greater than 1% for *BRAF* or *TERT* variants and cases in coexistence of two variants at any low or high VAFs.

© 2023 Fu G et al. *JAMA Network Open.*

3. eFigure

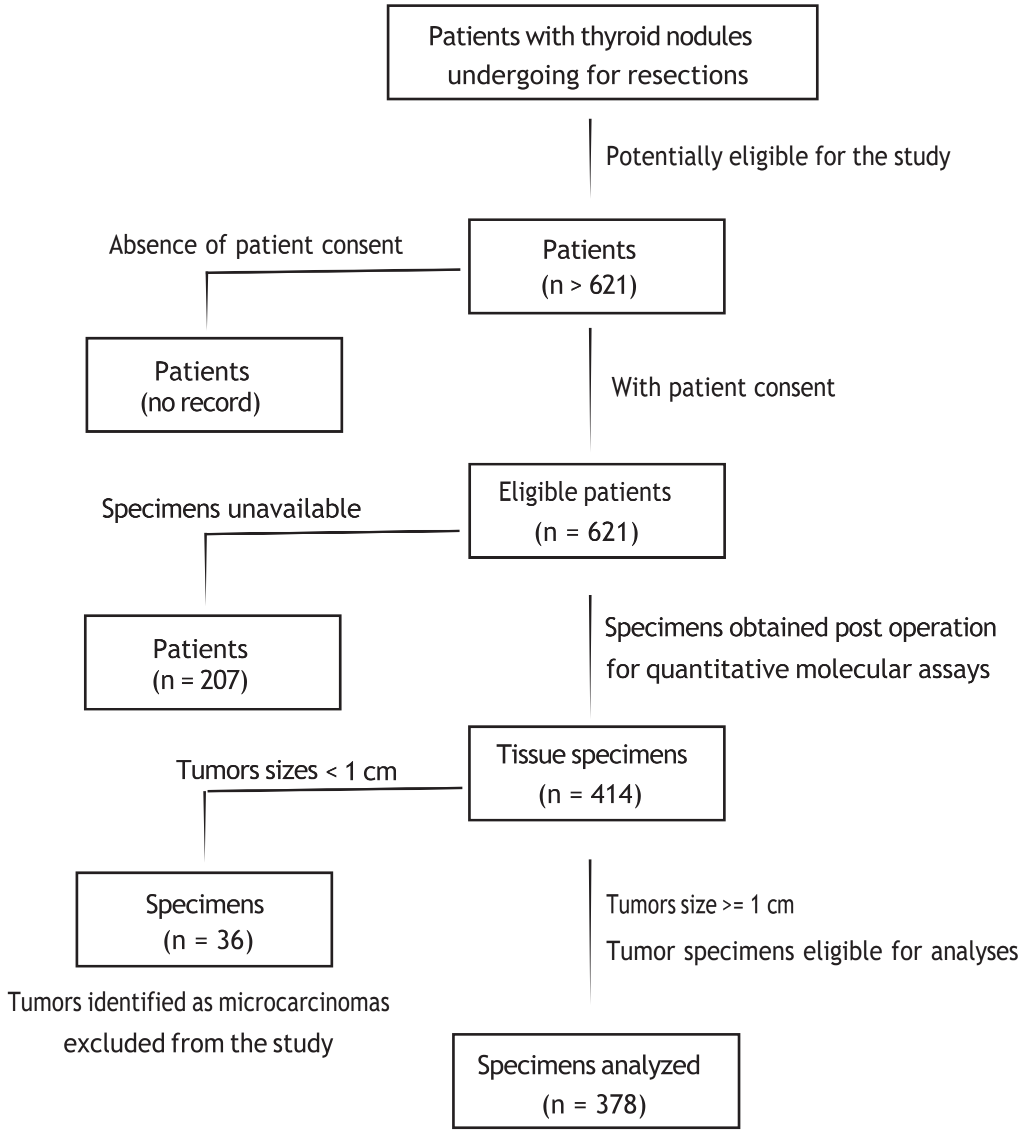

eFigure. Participant Flow Chart

The flow chart described the potentially eligible patient number identified between March 2016 and March 2020, the eligible number with patient's consent, and the number involved in the quantitative molecular assays and analyses in the current study. The numbers of individuals excluded from the study at each stage were shown due to the lack of patient's consent or no specimens available for research under certain conditions (For example, specimen was not obtained when a tumor was too small or invisible as it was reserved for histology in priority). Specimens were also excluded from analysis when tumors were subsequently identified as microcarcinomas.

© 2023 Fu G et al. *JAMA Network Open.*
