# Supplementary material for: Facilitation of Definitive Cancer Diagnosis With Quantitative Molecular Assays of BRAF V600E and TERT Promoter Variants in Patients With Thyroid Nodules
Source: JAMA Netw Open. 2023 Jul 28;6(7):e2323500. doi: 10.1001/jamanetworkopen.2023.23500 (PMC10383015; doi:10.1001/jamanetworkopen.2023.23500)
Supplement: Supplement 2. — Data Sharing Statement [file jamanetwopen-e2323500-s002.pdf]

Data Sharing Statement

Fu. Facilitation of Definitive Cancer Diagnosis With Quantitative Molecular Assays of BRAF V600E and TERT Promoter Variants in Patients With Thyroid Nodules. *JAMA Netw Open*. Published July 28, 2023. doi:10.1001/jamanetworkopen.2023.23500

Data
Data available: No

Additional Information
Explanation for why data not available: According to the existing patient consent we cannot make the data available to others or deposit it in public database without further approval by the Sinai Health Research Ethics Board. However, we are willing to share the data upon reasonable request and with fulfilled legal requirements (approval from all ethics committees and data transfer agreements).
